# ALS-FUS pathology revisited: singleton *FUS* mutations and an unusual case with both a *FUS* and *TARDBP* mutation

**DOI:** 10.1186/s40478-015-0235-x

**Published:** 2015-10-09

**Authors:** Andrew King, Claire Troakes, Bradley Smith, Matthew Nolan, Olimpia Curran, Caroline Vance, Christopher E. Shaw, Safa Al-Sarraj

**Affiliations:** Department Of Clinical Neuropathology, Academic Neuroscience Building, King’s College Hospital, Denmark Hill, SE5 9RS London, UK; MRC London Neurodegenerative Diseases Brain Bank, Institute of Psychiatry, Psychology and Neuroscience, King’s College London, SE5 8AF London, UK; King’s Health Partners Centre for Neurodegeneration Research, Department of Basic and Clinical Neurosciences, Institute of Psychiatry, Psychology and Neuroscience, Kings College London, SE5 8AF London, UK

## Abstract

**Introduction:**

Mutations in the *FUS* gene have been shown to be a rare cause of amyotrophic lateral sclerosis (ALS-FUS) and whilst well documented clinically and genetically there have been relatively few neuropathological studies.Recent work suggested a possible correlation between pathological features such as frequency of basophilic inclusions in neurons and rate of clinical decline, other studies have revealed a discrepancy between the upper motor neuron features detected clinically and the associated pathology. The purpose of this study was to describe the pathological features associated with more recently discovered *FUS* mutations and reinvestigate those with well recognised mutations in an attempt to correlate the pathology with mutation and/or clinical phenotype. The brains and spinal cords of seven cases of ALS-FUS were examined neuropathologically, including cases with the newly described p.K510E mutation and a case with both a known p.P525L mutation in the *FUS* gene and a truncating p.Y374X mutation in the *TARDBP* gene.

**Results:**

The neuropathology in all cases revealed basophilic and FUS inclusions in the cord. The density and type of inclusions varied markedly between cases, but did not allow a clear correlation with clinical progression. Only one case showed significant motor cortical pathology despite the upper motor neuron clinical features being evident in 4 patients. The case with both a *FUS* and *TARDBP* mutation revealed FUS positive inclusions but no TDP-43 pathology. Instead there were unusual p62 positive, FUS negative neuronal and glial inclusions as well as dot-like neurites.

**Conclusions:**

The study confirms cases of ALS-FUS to be mainly a lower motor neuron disease and to have pathology that does not appear to neatly correlate with clinical features or genetics. Furthermore, the case with both a *FUS* and *TARDBP* mutation reveals an intriguing pathological profile which at least in part involves a very unusual staining pattern for the ubiquitin-binding protein p62.

## Introduction

The discovery of the TAR DNA binding protein 43 (TDP-43) as a component of the neuronal inclusions in most cases of amyotrophic lateral sclerosis (ALS), and many cases of frontotemporal lobar degeneration (FTLD) marked a significant advance in the research into these devastating neurodegenerative diseases [1, 2]. Two years later another RNA binding protein termed fused in sarcoma (FUS) (also known as translated in sarcoma or TLS) was seen to be associated with a small number of ALS and FTLD cases, often in the young [3, 4]. Mutations in the *FUS* gene on chromosome 16, have been shown to be a rare cause of amyotrophic lateral sclerosis (ALS-FUS), being responsible for approximately 4 % of cases of familial ALS (fALS) and fewer than 1 % of sporadic cases [5–7]. While there have been a number of papers identifying the mutations associated with ALS-FUS, actual neuropathological descriptions of such cases are relatively sparse [8–12]. Those that have been described do detail FUS immunopositive inclusions in the anterior horn neurons of the spinal cord, instead of more classical TDP-43 inclusions. So far, however, unlike the TDP-43 proteinopathies there appears to be no obvious clinicopathological spectrum encompassing the cases of ALS with FUS (ALS-FUS), and the FTLD with FUS inclusions (FTLD-FUS). The two conditions seemingly have different pathophysiologies. Whereas ALS-FUS is associated with mutations and the inclusions contain exclusively FUS, FTLD-FUS is not usually associated with mutations and the inclusions appear to contain a number of so-called FET proteins including FUS but also transportin-1 [13–15]. ALS-FUS has an association with juvenile (i.e. younger than 25 years of age) ALS especially those showing abundant basophilic inclusions seen on H&E stains. Studies have attempted to group the pathological features of ALS-FUS into two distinct patterns, one correlating with younger age and more rapid clinical progression, the other with older age of onset and slower progression [8, 10]. Another publication with reference to genetic and clinical, but not pathological analysis, has indicated that truncating mutations in the FUS gene may give rise to a more aggressive phenotype than missense mutations [16].

The purpose of the current study was to both revisit and introduce pathological features of a series of cases of fALS with *FUS* mutations, (ALS-FUS) including mutations where the pathology has not to our knowledge been reported before, and also to illustrate the unusual pathology of a case with both a *FUS* mutation and *TARDBP* mutation. Attempts have also been made where possible to correlate genetic, clinical and pathological findings to test the assertion of there being discrete clinicopathological subgroups.

## Materials and methods

Brain and spinal cord tissue was examined from 7 cases of ALS with previously identified FUS mutations. Paraffin-embedded tissue blocks were obtained from the Medical Research Council London Neurodegenerative Diseases Brain Bank (Institute of Psychiatry, Psychology and Neuroscience King’s College London, UK). Consent for autopsy, neuropathological assessment and research was obtained from all subjects.

### Genetics

DNA was extracted from frozen post-mortem patient tissue with standard phenol-chloroform techniques, with one exception; which was to include a second chloroform step to ensure clean DNA free of protein contamination. As a point of thorough investigation, all cases were screened for the *C9ORF72* hexanucleotide GGGGCC expansion by repeat PCR as previously described [17], PCR of all exons of *SOD1* and the mutation clusters found in exon 6 of *TARDBP*, and exons 6,14 and 15 of *FUS*. PCR reactions for *SOD1*, *TARDBP* and *FUS* were confirmed by 2 % gel electrophoresis and products directly sequenced with Big-Dye® Terminator v1.1 on an ABI3130 genetic analyser (Applied Biosystems Pty Ltd, Warrington, UK). Sequence chromatograms were analysed for mutations by eye using Sequencher® 4.10 (Gene Codes Corporation, Ann Arbor, Michigan, USA). Identified mutations were reconfirmed by performing an independent PCR reaction from a fresh aliquot of stock DNA.

### Immunohistochemistry

Immunohistochemistry for FUS, p62 and phosphorylated TDP-43 (pTDP-43) was performed on (where available) the frontal lobe containing middle frontal gyrus, temporal lobe with superior and middle temporal gyrus, motor cortex (representing upper limb, lower limb, and face regions) in all except case 2 (no face region) and 3 (no face or lower limb regions), hippocampus, amygdala, basal ganglia, mid brain, medulla and cerebellum with dentate nucleus and spinal cord. Tau immunohistochemistry was performed on the frontal and temporal cortex, hippocampus and (where available) amygdala and immunohistochemistry for α-synuclein was performed on the medulla, pons, midbrain (and where available amygdala). β Amyloid antibody (Aβ) was applied to frontal and temporal neocortex and antibodies to CD68 were applied to the motor cortex. Sections of cord and medulla were cut at 14 μm thickness and stained for Luxol fast blue/Nissl. Otherwise the blocks were cut at 7 μm thickness and stained with haematoxylin and eosin (H&E). The relevant 7 μm thick sections were also immunohistochemically stained with the mouse monoclonal antibody to p62 (1:100; BD Biosciences, Erembodegem, Belgium), phosphorylated tau (clone [AT-8]; 1:500; Autogen Bioclear UK Ltd, Wiltshire, UK), α-synuclein (clone [42/α synuclein]; 1:500; Novocastra Laboratories Ltd, Newcastle-upon-Tyne, UK), CD68 (clone [PGM1]; 1:50; Dako, Glostrup, Denmark) and Amyloid β (Aβ) (1:12,000; Chemicon, Temecula, CA, USA), or the rabbit polyclonal antibody to phosphorylated pTDP-43 (pS409/410-2; 1:1500); Cosmo Bio Ltd, Tokyo, Japan), FUS (1:50; Proteintech Group, Chicago, USA) and one case only (case 6) non-phosphorylated (non p-)TDP-43 (1:400; Proteintech Group, Chicago, USA, and (41-7.1) 1:200; Santa-Cruz, Heidelberg, Germany) and additional FUS antibody (1:200; Sigma-Aldrich, Dorset, UK) using the Leica BONDMAX™ (Leica Biosystems, Wetzlar, Germany). Leica BONDMAX™ epitope retrieval sets was used for pTDP-43 and (non p-) TDP-43 (ER1 for 30 min), p62 and FUS (ER2 for 20 min,) CD68 and tau (ER1 for 20 min). For both α synuclein, and Aβ 80 % formic acid pre-treatment (for 1 h) was used. Nuclei were counterstained with Harris’ alum haematoxylin. For double labelling 7 μm sections were cut from formalin-fixed, paraffin-embedded blocks, dewaxed in xylene and dehydrated in 99 % industrial methylated spirit. Sections were then pretreated by microwaving in citrate buffer and blocked using normal goat serum (1:10 for 45 min). Primary antibodies against p62 and GFAP or p62 and FUS were then applied (p62, BD Biosciences, Erembodegem, Belgium 1:100: GFAP, DAKO, Glostrup, Denmark, 1:500; FUS, Sigma HPA00878, 1:100) and sections incubated at 4 °C overnight. Sections were washed and secondary Alexa Fluor antibody (goat anti-mouse 488 and goat anti-rabbit 568, Invitrogen, Paisley, UK) applied for 45 min (in dark). Autofluorescence was quenched by incubating the sections in Sudan black for 10 min followed by numerous washes in phosphate buffered saline before coverslip mounting using Vectashield hard set media with DAPI. Sections were visualised using a fluorescent microscope (Zeiss Axiovert S 100, Gottingen, Germany) and images captured using ImagePro Express (V6).

### Neuropathological analysis

The densities of the respective deposited proteins (TDP-43, p62, FUS) in neuronal/glial inclusions and neurites were scored semi-quantitatively (absent, small numbers/low density, moderate numbers/moderate density, large numbers/high density) in the areas specified above (the cervical level of the cord was selected for this analysis). The number of CD68 immunopositive microglial cells (absent, small numbers, moderate numbers, large numbers) was assessed in the motor cortex and neuronal loss in the spinal cord sections was also scored semi-quantitatively.

## Results

### Genetics

Six non-synonymous and one truncation *FUS* mutation were identified in seven familial ALS patients by direct sequencing. Mutations are located in the last 30 amino acids of the protein (residues 495–526, exons 14 and 15) that disrupt the nuclear localisation domain of the protein (Table 1). All have been previously described and are absent from either the NHLBI Exome Sequencing Project (evs.gs.washington.edu/EVS/) or the Exome Aggregation Consortium (exac.broadinstitute.org) [6]. All cases were negative for *C9ORF72* expansions, *SOD1* mutations and the mutation hotspot of *TARDBP* with one exception. Case 6 harboured both a p.P525L *FUS* mutation and also a *TARDBP* p.Y374X truncation mutation, the latter of which has been reported once previously in a French Male sporadic ALS patient with an age of onset of 63 years [18]. DNA was unavailable from either parent of case 6 to test for segregation of the p.P525L and p.Y374X mutations, however, clinical records describe the mother of case 6 to be a young onset ALS patient in which the early onset and aggressive course suggests the disease to be due to the *FUS* mutation.

**Table 1:** Summarising the FUS mutations and clinical features in the 7 cases

Case	Exon	∆Nucleotide	∆Protein	Age at death (yrs)	Sex	Duration (months)	UMN	LMN	Limb/Bulbar	Cog decline
1	14	c.1483C>T	p.R495X	34	F	9	Yes	Yes	L	No
2	14	c.1528A>G	p.K510E	39	M	15	Yes	Yes	B	No
3	15	c.1561C>T	p.R521C	33	M	10	NA	Yes	NA	No
4†	15	c.1561C>T	p.R521C	35	F	7	Yes	Yes	B	No†
5	15	c.1562G>A	p.R521H	35	F	>36	NA	Yes	L	No
6^a^	15	c.1574C>T	p.P525L^a^	23	F	8	Yes	Yes	L	No
7	15	c.1540A>G	p.R514G	60	M	>96	NA	Yes	B	No

Mutations identified in the gene encoding *FUS* (Refseq: NM_004960). cDNA location using +1 from the ATG start site

^a^Case 6 also carried a previously described p.Y374X *TARDBP* mutation (cDNA position c.1119_1120delTT). † Learning difficulties noted in clinical records

*Cog* Cognitive, *NA* Not available, *LMN* Lower motor neuron signs, *UMN* upper motor neuron signs

### Clinical

The clinical features are briefly summarised in Table 1. Cases 3, 4 and 5 have been previously described [4]. The mean age at death was 37 (range 23–60). The duration of time from onset of symptoms to death ranged from 7 months to more than 96 months. On reviewing the clinical notes available all 7 patients had lower motor neuron (LMN) symptoms some time in their disease process. Three cases had definite bulbar symptoms (3 no symptoms- and 1 unknown). Four cases had upper motor neuron (UMN) symptoms, with the remaining 3 unknown. None of the cases had any history of cognitive decline although case 4 was recorded as having learning difficulties since childhood.

### Neuropathology

**Fig. 1:**
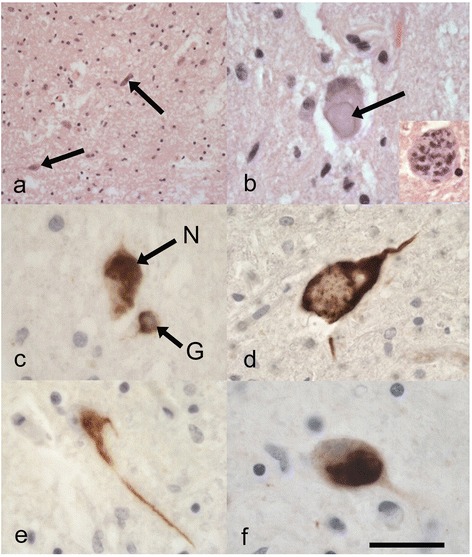
**a** Reveals marked neuronal loss in the anterior horn of the spinal cord with occasional surviving angulated neurons (*arrows*) in case 5 (p.R521H). H&E. **b** Well circumscribed basophilic inclusion (*arrow*) in an anterior horn neuron in case 6 (p.P525L and p.Y374X *TARDBP* mutation). This contrasts with an anterior horn neuron containing some basophilic aggregate-like inclusions (*inset*-case 2 (p.K510E)) which resemble condensed Nissl substance H&E. **c** Immunostaining for p62 illustrates neuronal (*N*) and glial cytoplasmic inclusions (*G*) in the anterior horns of the spinal cord. Case 1 (p.R495X). Anti p62. **d**-**f**. Reveal the differing shapes and conformations of FUS-positive neuronal cytoplasmic inclusions in the anterior horns of the spinal cords in case 1 (**d**-p.R495X), case 2 (**e**-p.K510E), and case 4 (**f**-p.R521C). Anti-FUS. *Scale Bar* (**a**)-140 μm, (**b**) (**c**) (**e**) and (**b**) *inset*-35 μm, (**d**) (**f**)-25 μm

**Fig. 2:**
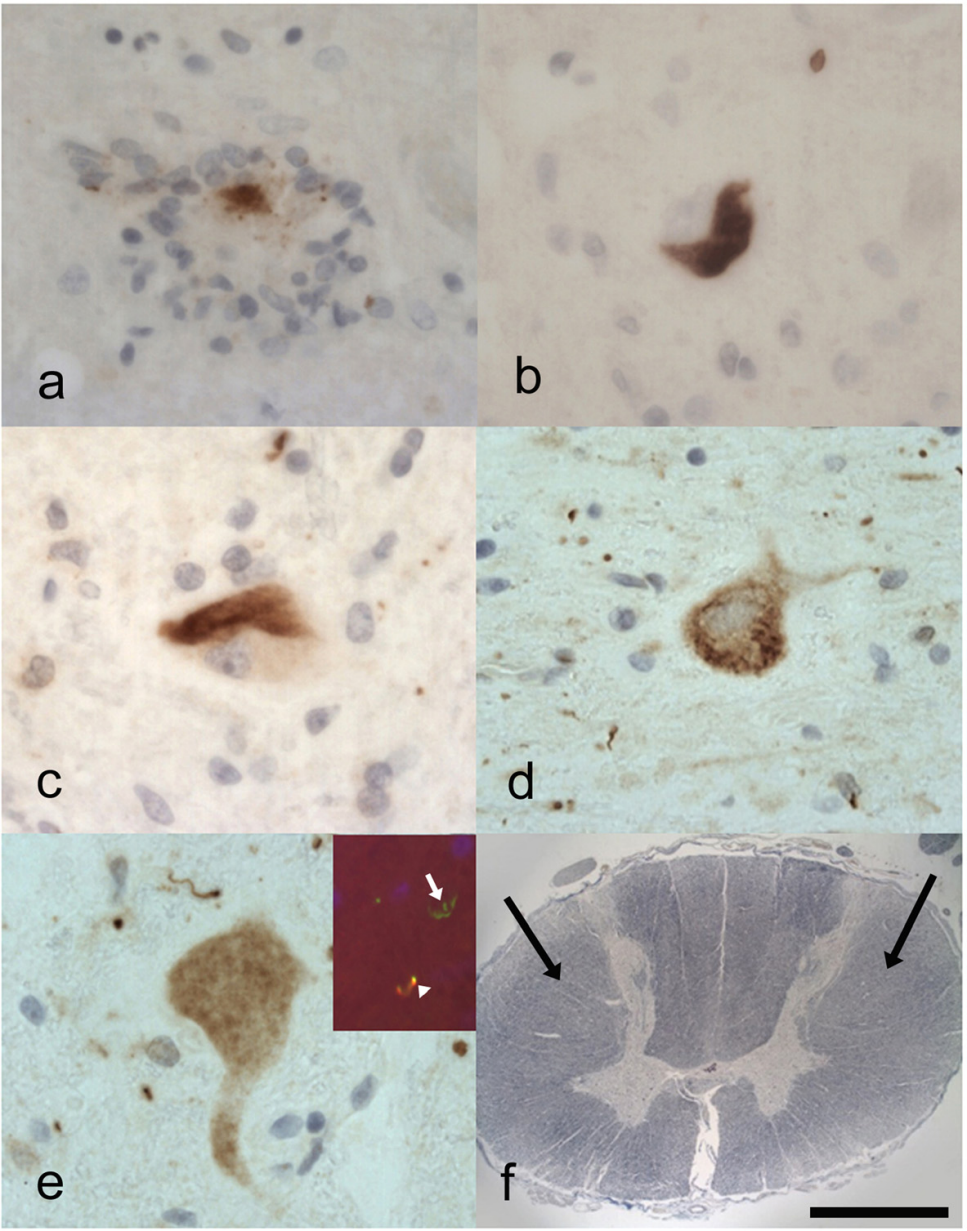
**a** Revealing a focus of neuronophagia in the anterior horn of the spinal cord in case 2 (p.K510E) with remnants of FUS immunopositivity. Anti-FUS. **b**-**e** Case 6 (p.P525L and p.Y374X *TARDBP* mutation) also does exhibit FUS-immunopositive neuronal cytoplasmic inclusions (**b**) in the anterior horns of the cord. These inclusions (**c**) are also immunopositive for p62. The p62 reveals additional neuronal positivity that does not appear to correspond to the FUS immunopositivity including more granular (**d**) and diffuse cytoplasmic staining (**e**). Double labelling for FUS (*red*) and p62 (*green*) in (**e**) *inset* shows co-localisation (*yellow*) in some neurons (*arrowhead*) but some p62 is not associated with FUS (*arrow*). Anti-FUS in (**b**). Anti-p62 in (**c**), (**d**) and (**e**). Anti-FUS and anti-p62 in (**e**) *inset*. **f** The cord in case 1 (p.R495X) revealing very mild loss of myelin in the lateral corticospinal tracts (*arrows*). LFB/N. *Scale Bar* (**a**)-(**c**) and (**e**)-30 μm, (**e**) *inset*-100 μm. (**d**)-40 μm, (**f**)-3000 μm

**Fig. 3:**
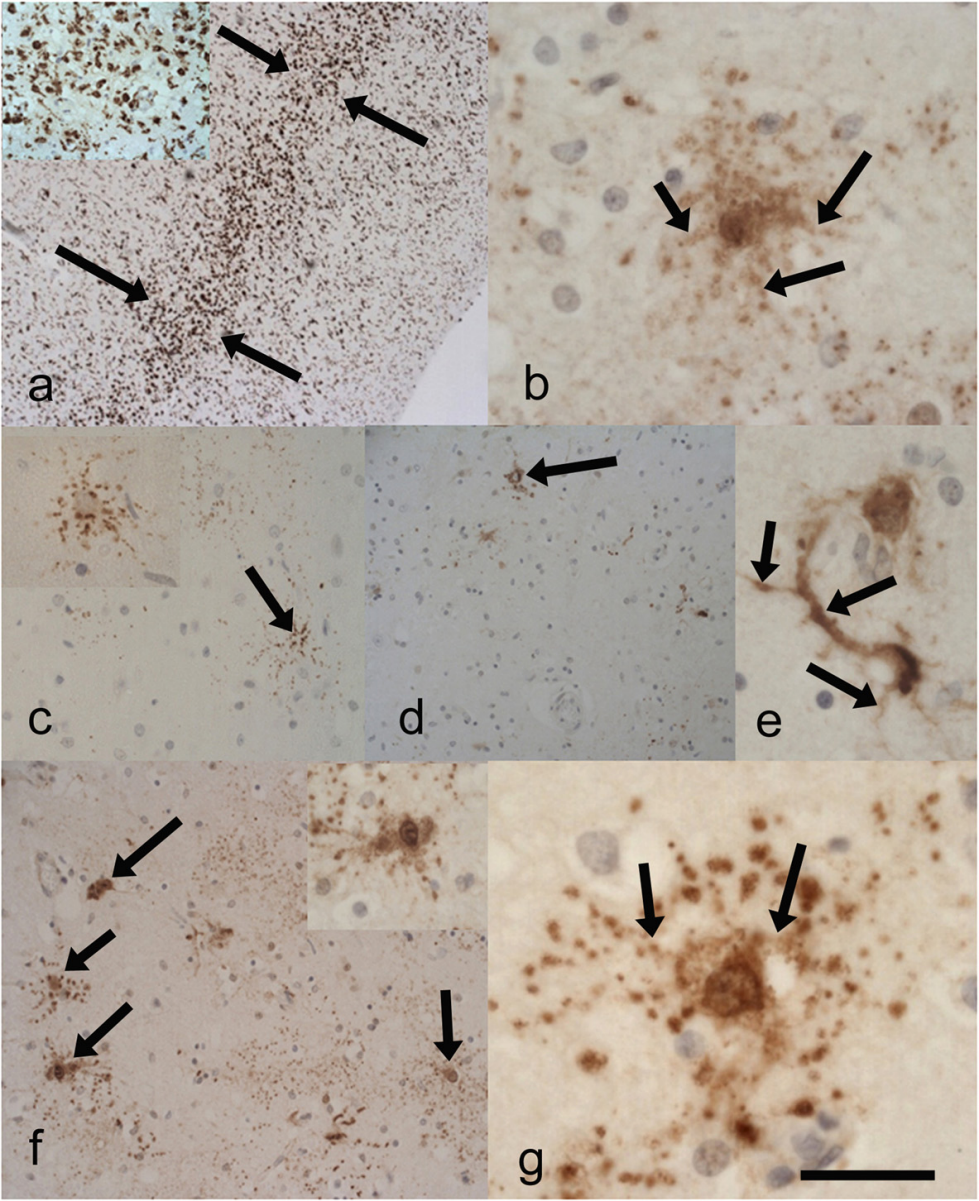
Case 6 (p.P525L and p.Y374X *TARDBP* mutation) (**a**) and *inset* illustrates marked microglial activity in the mid-deeper laminae (between *arrows*) of the motor cortex. Anti-CD68. **b** Showing the unusual neuronal staining for p62 in the motor cortex including processes (arrows). **c**-**e** revealing the sparse but unusual neuronal p62 immunopositivity in the hippocampal region (**c**) and (**c**) inset including occasional NCIs (*arrow*), and (**d**) the putamen revealing occasional NCIs (*arrow*). **e** Reveals one of the few p62 immunopositive neurons in the putamen, this is also apparently labelling the corresponding axon/dendrites (*arrows*) and appears different in pattern to the occasional FUS positive NCIs. P62 immunopositivity in the frontal neocortex (**f**) revealing dot-like positivity and neuronal inclusions (*arrows* and *inset*). **g** p62 immunopositive neuron in the temporal neocortex. These inclusions are negative for FUS and TDP-43 and appear to label the nuclear/ perinuclear region and processes (*arrows*) the latter indicating likely axonal/dendritic positivity. Anti-p62. *Scale Bar* (**a**)-300 μm, **a**-*inset* 100 μm (**b**)-50 μm, (**c**)-150 μm, (**c**) *inset*-80 μm, (**d**) 200 μm, (**e**)-50 μm, (**f**)-200 μm, (**f**) *inset*-80 μm, (g)-30 μm

The original pathological descriptions for cases 3, 4 and 5 have been previously described [4]. The neuropathological features of all seven cases are summarised in Table 2. *i) Spinal cord:* Five of the cases demonstrated severe neuronal loss within the anterior horn neurons of the spinal cord (Fig. 1(a)). The other 2 cases showed moderate loss. All of the seven cases revealed at least some basophilic inclusions in anterior horn neurons when viewed on H&E stains (Fig. 1(b)), however, the numbers of neurons with these inclusions varied between frequent and sparse (this may have been at least partially secondary to the degree of neuronal loss). Some of the inclusions were well circumscribed while others resembled condensed Nissl substance (Fig. 1(b) and inset). All the cases except case 3, showed p62 positive, FUS positive neuronal cytoplasmic inclusions (NCIs) in anterior horn cells (Fig. 1(c)-(f)) (case 3, did show FUS positive glial cytoplasmic inclusions (GCIs) in the cord). These FUS-positive NCIs when present, were moderate (or moderate-frequent) in numbers apart from case 7 where there were relatively few. Some appeared to correspond to the basophilic inclusions. Occasional foci of neuronophagia were evident (Fig. 2(a)). All the cases revealed FUS immunopositive GCIs in the cord, but these varied from mild to high densities. In all cases except 6, the p62 immunohistochemistry approximately corresponded to the FUS immunohistochemistry (Case 3 showed slightly more p62 positivity). Case 6 showed FUS immunopositivity (Fig. 2(b)) and more marked p62 positivity in glial and neuronal cells. The p62 not only labelled the type of NCIs that were immunopositive for FUS (Fig. 2(c)), but there were some additional NCIs with more granular and diffuse cytoplasmic staining (Fig. 2(d) and (e)). Double labelling indeed revealed some neurons positive for both p62 and FUS, whilst others were only p62 positive (Fig. 2(e inset)). In all cases the LFB/N stain showed minimal-very mild loss of myelin over the lateral corticospinal tracts (Fig. 2(f)). *ii) Medulla:* All except case 4 showed FUS positive NCIs in the twelfth nerve nucleus (this case had FUS positive GCIs in this region). *iii) Motor cortex:* Betz cells were seen in all sections. Apart from case 6, the motor cortex revealed mild changes with little evidence of neuronal loss or microglial proliferation (as judged by CD68 immunohistochemistry). All cases except case 3 showed FUS positive NCIs in this region. Case 6 showed marked microglial proliferation in the motor cortex (Fig. 3(a)), and whilst there were only occasional FUS-positive NCIs and GCIs present in the motor cortex (confirmed by 2 FUS antibodies) the p62 revealed much more extensive immunopositivity. This was in the form of intense granular cytoplasmic staining in neurons and dots in the adjacent neuropil which may have reflected affected axons or dendrites (Fig. 3(b)). Glial cells also showed positivity, although double labelling only showed occasional GFAP positive astrocytes co-expressing p62. *iv) Other brain regions:* Of the six cases where basal ganglia were sampled all except case 1, showed some FUS positive NCIs or GCIs. All cases except 4, 5, and 7 revealed FUS positive NCIs in the substantia nigra. No case showed FUS immunopositivity in the hippocampus, amygdala, frontal neocortex or temporal neocortex. No laminar cortical necrosis was seen. Only cases 2 and 4 showed FUS positivity (neuronal or glial) in the dentate nucleus of the cerebellum.

**Table 2:** A summary of the pathological features in the 7 cases of ALS-FUS

Case	Frontal Cx	Temp Cx	Hippo	BG Put/GP	Amy	Motor	Mid brain Nigra	Medulla XII	Spinal cord	Other
1	FUS -ve	FUS -ve	FUS -ve	FUS -ve	FUS -ve	FUS/p62	FUS/p62:	FUS:	Ant horn: Moderate neuronal loss.	
	TDP -ve	TDP -ve	TDP -ve	TDP -ve	TDP -ve	NCI +	NCI +	NCI +	Numerous basophilic inclusions	
	p62 -ve	p62 -ve	p62 -ve	p62 –ve	p62-ve	GCI -ve	GCI +	GCI ++	FUS/p62 : NCI ++, GCI +++	
						TDP-ve	TDP-ve	P62:		
						CD 68 +		NCI +	TDP -ve	
								GCI +		
								TDP-ve	Lat. c-spinal tracts myelin loss: very mild	
2	FUS -ve	FUS -ve	FUS -ve	FUS/p62	FUS -ve	FUS:	FUS/p62:	FUS/p62:	Ant horn: Moderate neuronal loss.	Mild FUS in cerebellar dentate
	TDP -ve	TDP -ve	TDP -ve	NCI ++	TDP -ve	NCI +	NCI ++	NCI ++	Moderate basophilic inclusions	
	p62 -ve	p62 -ve	p62 -ve	GCI++	p62 –ve	GCI +	GCI ++	GCI +++	FUS: NCI ++, GCI ++	
				TDP-ve		P62:	TDP-ve	TDP-ve	p62: NCI +, GCI ++	
						NCI -ve				
						GCI +			TDP -ve	
						TDP –ve				
						CD68 +			Lat. c-spinal tracts myelin loss: mild	
3	FUS -ve	FUS -ve	FUS -ve	NA	NA	FUS/p62:	FUS/p62:	FUS:	Ant horn: Severe neuronal loss.	
	TDP -ve	TDP -ve	TDP -ve			-ve		NCI +	Moderate basophilic inclusions	
	p62 -ve	p62 -ve	p62 -ve			TDP -ve	NCI +	GCI -ve	FUS: NCI -ve, GCI +	
						CD68 +	GCI +	P62:	p62: NCI +, GCI +	
							TDP-ve	NCI -ve		
								GCI +	TDP -ve	
								TDP-ve	Lat. c-spinal tracts myelin loss: very mild	
4	FUS -ve	NA	FUS -ve	FUS:	FUS -ve	FUS:	FUS -ve	FUS:	Ant horn: Severe neuronal loss.	Mild FUS in cerebellar dentate
	TDP--ve		TDP -ve	NCI+	TDP -ve	NCI +	p62:	NCI -ve	Moderate basophilic inclusions	
	p62 -ve		p62 -ve	GCI ++	p62 -ve	GCI +	NCI +	GCI +	FUS: NCI ++, GCI ++	
				p62:		P62:	GCI ++	P62:	p62: NCI ++, GCI +++	
				NCI++		GCI ++	TDP-ve	NCI +		
				GCI++		TDP-ve		GCI +++	TDP -ve	
				TDP-ve		CD68 +		TDP-ve		
									Lat. c-spinal tracts myelin loss: very mild	
5	FUS -ve	FUS -ve	FUS -ve	FUS:	FUS -ve	FUS/p62:	FUS:	FUS:	Ant horn: Severe neuronal loss.	
	TDP -ve	TDP -ve	TDP -ve	NCI +	TDP -ve	NCI +	NCI -ve	NCI ++	Occasional basophilic inclusions	
	p62 -ve	p62 -ve	p62 -ve	GCI+	p62 -ve	GCI +	GCI +	GCI ++	FUS: NCI ++, GCI ++	
				P62-ve		TDP-ve	P62:	P62:	p62: NCI +, GCI +++	
				TDP-ve		CD68 +	NCI +	NCI -ve		
							GCI +	GCI ++	TDP -ve	
							TDP-ve	TDP-ve		
									Lat. c-spinal tracts myelin loss: mild	
6	FUS -ve	FUS -ve	FUS -ve	FUS:	FUS -ve	FUS:	FUS/p62:	FUS:	Ant horn: Severe neuronal loss.	
	TDP -ve	TDP –ve	TDP –ve	NCI +	TDP -ve	NCI +		NCI ++	Moderate basophilic inclusions	
				GCI+	P62:	GCI +	NCI +	GCI ++	FUS: NCI ++, GCI ++	
	p62 :	p62 :	p62 :	p62:	NCI +	P62:	GCI +	P62:	p62: NCI +++, GCI +++	
	NCI +++	NCI +++	NCI +	NCI+	GCI +	NCI++	TDP-ve	NCI ++		
	GCI+++	GCI+++	GCI+	GCI++		GCI++		GCI +++	TDP -ve	
	Neur +++	Neur ++	Neur ++	TDP-ve		TDP-ve		TDP-ve		
						CD68 +++			Lat. c-spinal tracts myelin loss: very mild	
7	FUS -ve	FUS -ve	FUS -ve	FUS:	FUS -ve	FUS/p62:	FUS:	FUS:	Ant horn: Severe neuronal loss.	TAU + in hippo
	TDP--ve	TDP -ve	TDP -ve	NCI ++	TDP -ve	NCI +	NCI -ve	NCI ++	Occasional basophilic inclusions	
	p62 -ve	p62 -ve	p62 -ve	GCI++	P62:	GCI +	GCI +	GCI +	FUS: NCI +, GCI ++	
				p62:	NCI -ve	TDP-ve	P62:	P62:	p62: NCI +, GCI +	
				NCI -ve	GCI +	CD68 +	NCI +	NCI +		
				GCI +			GCI –ve	GCI +++	TDP -ve	
				TDP-ve			TDP -ve	TDP-ve		
									Lat. c-spinal tracts myelin loss: mild	

*Amy* Amygdala, *Ant* anterior, *BG* Basal ganglia, *C-spinal* Corticospinal, *Cx* Cortex, *GCI* Glial cytoplasmic inclusions, *GP* globus pallidus, *Hippo* Hippocampus, *Lat* lateral, *NA* Not available, *NCI* Neuronal cytoplasmic inclusions, *Neur* Neurites, *Put* Putamen, *TDP* TDP-43, *XII* XIIth nerve nucleus, *-ve* Not present, + small numbers/low density, ++ moderate numbers/moderate density, +++ large numbers/high density

*v) Unusual p62 staining pattern:* Case 6 showed p62 positivity in the hippocampus (but not the dentate gyrus), mainly in the form of neurites with only small numbers of NCIs (Fig. 3(c)), there was also mild p62 positivity in the amygdala and basal ganglia (Fig. 3(d)-(e)). The p62 immunopositivity in the frontal and temporal neocortex was similar in appearance to that in the motor cortex with ramifying and granular NCIs (some with additional nuclear/perinuclear staining), GCIs and dot-like neuropil positivity (Fig. 3(f) and (g)). *vi) Other proteins*: There was minimal tau staining in case 7 in the hippocampus, entorhinal cortex and amygdala. No other case showed any staining for tau. The staining for Aβ, α-synuclein and pTDP-43 was completely negative in all cases, including case 6. In addition, case 6 revealed a normal staining pattern for non-phosphorylated TDP-43 (using 2 antibodies) despite having a *TARDBP* mutation. Specifically there was no loss of normal nuclear staining or cytoplasmic inclusions identified.

## Discussion

Fused in sarcoma (FUS) mutations on chromosome 16 have in recent years been recognised as a relatively rare, yet important cause of amyotrophic lateral sclerosis (ALS). In all, such mutations represent approximately 4 % of familial ALS cases (fALS) and less than 1 % of sporadic cases. The FUS protein is thought to have, amongst other functions, RNA binding properties similar to TDP-43, which itself is associated with the majority of ALS cases (both sporadic and familial) [19]. When investigated neuropathologically cases of ALS-FUS have been shown to exhibit FUS immunopositive inclusions in the anterior horn neurons of the spinal cord, the twelfth cranial nerve nucleus of the medulla, and/or within the neurons of the motor cortex [8–12]. The distribution and shape of the neuronal cytoplasmic inclusions (NCIs) have some similarities to those seen in ALS associated with deposition of TDP-43 (ALS-TDP). These NCIs can be filamentous-like and more globular, sometimes they are also associated with glial cytoplasmic inclusions (GCIs) and/or neurites. ALS associated with TDP-43 positive inclusions (ALS-TDP) and frontotemporal lobar degeneration with TDP-43 positive inclusions (FTLD-TDP) (a pathological process associated with frontotemporal dementia, semantic dementia and/or progressive non fluent aphasia) are believed to represent a clinicopathological spectrum, where some patients have clinical and pathological features of a frontotemporal dementia and ALS (FTLD-MND/ALS). ALS-FUS, however, appears to be distinct both clinically and pathologically from the frontotemporal lobar degeneration with FUS positive inclusions (FTLD-FUS) [13-15, 20]. Many cases of ALS-TDP, even when not associated with cognitive decline, still show TDP-43 immunopositive inclusions in the limbic regions such as hippocampus, amygdala or entorhinal cortex. Our study confirmed previous reports in that, apart from some inclusions in the basal ganglia and cerebellum, there were no cases with extramotor FUS positive inclusions, further confirming the distinction between ALS-FUS and FTLD-FUS. Indeed FTLD-FUS is not usually associated with mutations. Instead FTLD-FUS, unlike ALS-FUS, appears to exhibit inclusions that contain a number of so-called FET proteins including FUS, Ewing’s sarcoma protein (EWS) and TATA-binding protein associated factor 15 (TAF15) as well as Transportin 1 implying abnormalities in nuclear-cytoplasmic transport [13, 15, 21]. Additionally, in ALS-FUS mutations the arginine methylation status of FUS appears to differ from that in FTLD-FUS [14, 20, 22, 23]. Indeed the ALS-FUS mutations may instead result in the production of a more rigid protein which in turn appears to affect its RNA binding properties [19, 24].

Combined with previous work, this study suggests that in the early stages of ALS-FUS most *FUS* mutations preferentially exert their effects on the spinal motor neurons [11]. Whilst there have been studies attempting to correlate particular *FUS* mutations with clinical progression, there have been relatively few descriptions of pathology in ALS-FUS. Waibel et al. found that cases of ALS-FUS with truncating mutations were associated with a more aggressive phenotype than missense mutations [16]. This cannot be confirmed or refuted in this series because only one truncating mutation case is present, however, even 2 of the missense mutations had rapid disease progressions of 10 months or less. One pathological feature of particular interest is that all cases showed only mild to very mild loss of myelin in the lateral corticospinal tracts. This was in contrast to 4 of the cases demonstrating at least some UMN features clinically and one case (case 6) showing evidence of marked microglial activity in the motor cortex. Previous studies have also demonstrated this discrepancy; the clinical presentation in ALS-FUS is often of early proximal limb weakness sometimes with pelvic or scapular weakness, mild or late-onset bulbar features and no significant cognitive impairment and mild or no extrapyramidal abnormalities [10, 25]. Despite this, in Mackenzie et al.’s series the paucity of UMN features did not appear to correlate with the relatively severe FUS pathology in the motor cortex. Similarly Baumer et al. illustrated 2 cases with the p.P525L mutation, one of whom had no UMN features clinically yet marked degeneration of the corticospinal tracts [8]. This was further illustrated in a study by Riku et al. who attempted to distinguish the clinicopathological features of the lower motor syndrome of progressive muscular atrophy (PMA) with typical clinical ALS [26]. They found (albeit with relatively small numbers) that whilst cases of ALS-FUS accounted for 15 % of the PMA cases, all of the typical clinical ALS cases showed TDP-43 pathology. Nevertheless, in these 15 % (which were represented by 2 cases) there were still FUS positive inclusions seen in the motor cortex. In their series of 6 cases (4 with FALS) Hewitt et al. described minimal FUS pathology in the motor cortex [9]. However, there have been reported exceptions with one patient suffering from a locked-in state associated with a p.K510M mutation and abundant FUS pathology in the UMNs [27]. Tateishi et al. described a multiple system degeneration associated again with marked UMN pathology but this time in patients with a p.R521C mutation which is identical to our cases 3 and 4 and thus illustrates the degree of phenotypical variability [28]. Mouse models with *FUS* mutations tend to support the hypothesis that it is the LMNs which are particularly vulnerable to the structural changes that these mutations cause [29]. All of our cases exhibited some basophilic inclusions in the anterior horn neurons of the cord. Mackenzie et al. attempted to define specific groups of ALS-FUS by pathological patterns including the density of basophilic inclusions [10]. Those with numerous basophilic inclusions (including 2 cases with p.P525L mutations) tended to have a more rapid disease progression and appeared to have fewer GCIs, compared to cases with fewer basophilic inclusions and more glial inclusions who had a later onset, and slower progression (including 2 cases with p.R521C mutations). Our findings do not fit neatly into this distinct grouping. Whereas case 5 with a p.R521H mutation would certainly appear to partially conform to the above criteria with a disease progression of at least 36 months, and associated with few basophilic inclusions in the cord, there were, however, moderate numbers of GCIs. Similarly case 7 had few basophilic inclusions in the cord, and also a relatively slow progression but again moderate numbers of GCIs. An explanation for this discrepancy might be due to a much younger-aged cohort in Mackenzie et al.’s group, or alternatively in our group the reduced numbers of basophilic neurons may just have been a reflection of the severe neuronal loss present in the cord. Case 6 (with a p.P525L mutation) also had moderate-large numbers of glial inclusions, yet also had moderate numbers of basophilic inclusions but a rapid disease progression. However, there is an additional element in case 6 which may have affected this, in that this patient as well as having a p.P525L mutation also had a truncating p.Y374X *TARDBP* mutation on chromosome 1. The pathology in this case 6 was very unusual. Whilst there was moderate FUS pathology in the form of NCIs and GCIs in the spinal cord, the twelfth nerve nucleus, and mild pathology for FUS in the motor cortex, there was marked CD68 positivity in the motor cortex indicating increased microglial activity and associated with neuronal loss in this region. There was no TDP-43 pathology seen, but there was extensive p62 pathology, in the form of granular and synaptic-like positivity in the cytoplasm and cell membrane of neurons and glial cells together with extensive dot-like and neurite-like neuropil positivity. Furthermore, this was not only seen in the cord but also even more extensively in the motor cortex and neocortex where there was a more ramifying pattern to the staining in the neurons . This *FUS* mutation has been described previously but not with this associated p62 pathology [8, 10]. Daoud et al. have described the genetics of the p.Y374X *TARDBP* mutation and have predicted this to be a damaging variant, but as far as is known there have been no pathological descriptions [18]. P62 also known as sequestosome 1 is a ubiquitin-binding protein, important in protein degradation via the ubiquitin-proteasome system. As such it often can be demonstrated in association with, and sometimes co-localised with, abnormal proteins in specific neurodegenerative diseases such as tangles in Alzheimer’s disease and Lewy bodies in Parkinson’s disease. It is also seen in association with TDP-43 in FTLD-TDP and ALS associated with TDP-43. It is intriguing that despite the *TARDBP* mutation in case 6 there was no TDP-43 pathology. Whereas this patient died at the age of 23, the only known reported patient with the same *TARDBP* mutation died at the age of 63 so it may simply be that the *FUS* mutation is the more “aggressive” mutation and they act independently such that we are seeing the end stage of the FUS-ALS disease and the earliest (if any) stage of the ALS-TDP disease [18]. This, however, does not adequately explain the unusual p62 expression. Daoud et al. predicted that the actual *TARDBP* mutation produces a truncated TDP-43 protein due to a premature stop codon removing the last 41 amino acids [18]. It would therefore be logical that any inclusions could not be detected with the relatively specific antibody to pTDP-43. What is more difficult to explain is that the antibodies to the wild type (non-phosphorylated) TDP-43 also showed no inclusions and indeed no loss of normal nuclear TDP-43 immunopositivity. Homma et al. have described a missense *TARDBP* mutation with minimal TDP-43 pathology [30], and it may be that the TDP-43 pathology in our case is also so subtle it has been missed. It is also possible that the mutation resulted in unusual species of TDP-43 not detectable by conventional antibodies. Although not directly comparable with a truncating mutation it is interesting that mouse models that have undergone conditional gene depletion of *TARDBP* in the spinal cord neurons do not show TDP-43 positive inclusions (unlike missense mutation models) but do show an unusual pattern of polyubiquitinated proteins in the neurons but not in the form of the usual inclusions [31–33]. Therefore in this case the p62 expression may be showing 2 distinct patterns, one simply shadowing the FUS pathology and one corresponding to an unusual pattern of polyubiquitinated proteins similar to the *TARDBP* depletion model described. Whilst the p62 pathology may indicate damage or dysfunction in individual neurons or glial cells the relationship cannot be that straightforward since the intensity of such p62 immunopositivity in the neocortex does not appear to correlate with cognitive decline. In this regard it can perhaps be compared to the extensive p62 and ubiquitin immunopositivity seen in the cerebellum in cases of FTLD or ALS with the *C9ORF72* repeat expansion. The immunoexpression here again does not appear to be related to neuronal loss or cerebellar signs, however, there is still a debate as to whether there is a toxic effect of the offending ubiquitinated dipeptide repeat proteins [34–36]. There also remains the theoretical possibility that there is a third mutation present (in the p62/sequestosome 1 (SQSTM1) gene) in this case which has given rise to the p62 pathology [37].

As far as is known this is the first pathological description of a patient with ALS-FUS associated with a p.K510E mutation. Syriani et al. described the clinical and genetic features of 2 cases with this mutation, one was 32 years old at death the other 43, and both had a rapid clinical course (8 and 10 months respectively), and neither demonstrated UMN signs [38]. Our case (case 2) did demonstrate some UMN and bulbar signs and a moderately rapid disease progression of 15 months. Pathologically what was interesting was the moderate to severe FUS involvement of the cord and medulla but again not the motor cortex. Also there were moderately numerous FUS positive NCIs and GCIs in the midbrain and basal ganglia.

The p.R514G missense mutation again has been detected previously and has been predicted to have a relatively mild phenotype [16], which is in keeping with our case 7, again to our knowledge the pathology associated with this mutation has not been reported. There were few basophilic inclusions and moderate numbers of FUS positive GCIs.

## Conclusions

In conclusion, our study has revealed the neuropathological features associated with both some well-known and more recently discovered *FUS* mutations. It has supported previous studies which have demonstrated that ALS-FUS is predominantly a lower motor neuron disease, it has also indicated that there can be marked clinical and pathological variability even between patients with identical mutations. Accordingly, at least in our cohort, the relationship between the density of basophilic inclusions in neurons, density of GCIs and clinical course in ALS-FUS is not straightforward. Furthermore, the study characterises the pathology of a patient with both a *FUS* and *TARDBP* mutation. The possible interaction of 2 pathological processes is intriguing but before it can be understood more neuropathological data will be required on cases with the rare unusual truncating *TARDBP* mutation, and any other rare combinations of *TARDBP* and *FUS* mutations. Alternatively a mouse model incorporating both would provide some answers regarding the relative contributions of the two mutations to the disease pathogenesis and the normal intracellular roles and interactions of both TDP-43 and FUS.
